# Complete Genome Sequence of Beak and Feather Disease Virus Isolated from an African Grey Parrot in China in 2017

**DOI:** 10.1128/genomeA.01519-17

**Published:** 2018-02-08

**Authors:** Xuejun Guo, Xinna Ge, Jianyu Chang

**Affiliations:** a Key Laboratory of Animal Epidemiology of the Ministry of Agriculture, College of Veterinary Medicine, China Agricultural University, Beijing, People's Republic of China

## Abstract

The complete nucleotide (nt) sequence of beak and feather disease virus (BFDV) was determined. The viral genome consists of 1,991 nt, including an 870-nt open reading frame 1 (ORF1), a 744-nt ORF2, a conserved stem-loop structure, and the second hairpin. This is the first reported detection of BFDV in an infected African grey parrot in China.

## GENOME ANNOUNCEMENT

Beak and feather disease virus (BFDV) is classified under the genus *Circovirus* of the family *Circoviridae*. The viral genome is a circular single-stranded DNA with a size of approximately 2 kb and contains two major open reading frames (ORFs) (ORF1 and ORF2) that encode the viral replication-associated protein Rep and the major structural capsid protein CP. BFDV is specific for members of the *Psittacidae* and causes psittacine beak and feather disease (PBFD), which is characterized by abnormally shaped feathers and beak in chronic forms and sudden death in acute forms (1–3). BFDV has been found worldwide and has been detected in a large variety of bird species (4–6).

In this study, fresh liver tissues were collected from dead African grey parrots in Beijing in China in 2017. Viral DNA was extracted, and the overlapping segments of the PBFDV genome were amplified by PCR with the use of two pairs of primers as previously described. PCR conditions were optimized as follows: 94°C for 2 min, followed by 35 cycles of 94°C for 30 s; 55°C for 30 s; and 72°C for 3 min (7). The nucleotide sequences were constructed by using the Geneious software DNAMAN.

The complete genome sequence of the PBFDV2017 isolate was 1,991 nucleotides (nt) in length, with a GC content of 52.94%. The ORF1 encoding Rep and ORF2 encoding CP were 870 nt and 744 nt in length, respectively. The most conserved nonanucleotide motif was TAGTATTAC, which was positioned at approximately nt 1 of the sequence located at the apex of a stem-loop structure in the nontranslated intergenic region. An exceedingly GC-rich (96.6%) segment of 29 nt in length was found at approximately nt 1130. The second hairpin was located from nt 13 to nt 57, the same location as in *Psittacidae erithacus* PEG07-1GE (GenBank accession no. AY521237), and had a single-base deletion at nt 1196 (1). BLAST results showed that the PBFDV isolate obtained in this study was related most closely to strain BFDV7 isolated in Thailand (GenBank accession no. FJ685979), with a 97.6% nucleotide identity rate and a single-base deletion.

In conclusion, the reported PBFDV2017 sequence was derived from an infected African grey parrot in Beijing in China. The affected bird lost contour feathers over most areas of the body before it died, suggesting that the PBFDV strain was highly pathogenic. Only one isolate of BFDV from budgerigars in China has been described, which was reported prior to 2013 (7), and the nucleotide identity rate between this isolate and the isolate we report here is 84.7%. Our results suggest the necessity for further epidemiological investigation and development of control strategies against this disease.

### Accession number(s).

The whole-genome sequence of PBFDV2017 has been deposited in GenBank under the accession no. MG257487.

## References

[1] de Kloet E, de Kloet SR 2004 Analysis of the beak and feather disease viral genome indicates the existence of several genotypes which have a complex psittacine host specificity. Arch Virol 149:2393–2412. doi:10.1007/s00705-004-0368-x. 15290360

[2] Pass DA, Perry RA 1984 The pathology of psittacine beak and feather disease. Aust Vet J 61:69–74. doi:10.1111/j.1751-0813.1984.tb15520.x. 6743145

[3] Ogawa H, Chahota R, Ohya K, Yamaguchi T, Fukushi H 2013 Relatedness between host species and genotype of beak and feather disease virus suggesting possible interspecies cross infection during bird trade. J Vet Med Sci 75:503–507. doi:10.1292/jvms.12-0367. 23149463

[4] Sarker S, Lloyd C, Forwood J, Raidal SR 2016 Forensic genetic evidence of beak and feather disease virus infection in a powerful owl, *Ninox strenua*. Emu 116:71–74. doi:10.1071/MU15063.

[5] Sarker S, Moylan KG, Ghorashi SA, Forwood JK, Peters A, Raidal SR 2015 Evidence of a deep viral host switch event with beak and feather disease virus infection in rainbow bee-eaters (*Merops ornatus*). Sci Rep 5:14511. doi:10.1038/srep14511. 26411487 PMC4585972

[6] Hakimuddin F, Abidi F, Jafer O, Li C, Wernery U, Hebel Ch, Khazanehdari K 2016 Incidence and detection of beak and feather disease virus in psittacine birds in the UAE. Biomol Detect Quantif 6:27–32. doi:10.1016/j.bdq.2015.10.001. 27077045 PMC4822206

[7] Zhuang Q, Chen J, Mushtaq MH, Chen J, Liu S, Hou G, Li J, Huang B, Jiang W 2012 Prevalence and genetic characterization of avian polyomavirus and psittacine beak and feather disease virus isolated from budgerigars in mainland China. Arch Virol 157:53–61. doi:10.1007/s00705-011-1138-1. 22002652

